# Beneficial effects of American ginseng (*Panax quinquefolius* L.) extract residue as a feed additive on production, health status, and gastrointestinal bacteria in sika deer (*Cervus nippon*)

**DOI:** 10.3389/fmicb.2024.1344905

**Published:** 2024-03-13

**Authors:** Yan Wu, Shuting Zhao, Peihe Zheng, Hanlu Liu, Zhengyi Qu, Wei Hou, Weitao Yuan, Tao Feng, Xiaofeng Zhan, Jinlong Shen, Kaiying Wang

**Affiliations:** ^1^Innovation Center for Feeding and Utilization of Special Animals in Jilin Province, Research Center for Microbial Feed Engineering of Special Animals in Jilin Province, Institute of Special Animal and Plant Sciences, Chinese Academy of Agricultural Sciences, Changchun, China; ^2^Jilin Agricultural Science and Technology University, Jilin City, China; ^3^Key Laboratory of Agro-ecological Protection and Exploitation and Utilization of Animal and Plant Resources in Eastern Inner Mongolia, Chifeng University, Chifeng, China; ^4^Hongjiu BioTech Co., Ltd., Tonghua, China; ^5^Jilin Province Shuangyang Deer Industry Stock Breeding Co., Ltd., Changchun, China

**Keywords:** American ginseng residue, antioxidant, gastrointestinal bacteria, rumen fermentation, sika deer

## Abstract

American ginseng residue is an industrial by-product of ginseng saponin extraction, including polysaccharides and amino acids; however, it is often discarded into the natural environment, representing a waste of resources as well as an environmental issue. In this study, we examined the effects of adding American ginseng residue to the basal diet of sika deer. Twelve antler-bearing male sika deer were assigned randomly to groups fed a diet supplemented with 0% (CON), 1% (LGR), and 3% (HGR) American ginseng residue, respectively, (*n* = 4 per group) for 5 weeks. Supplementation with 3% American ginseng residue significantly increased antler production and feed utilization efficiency in antler-bearing sika deer (*p* < 0.05). There were no significant differences in serum biochemical indexes among the three groups, but serum immunoglobulin A and glutathione peroxidase levels were significantly increased in the LGR and HGR groups (*p* < 0.05). Supplementation with American ginseng residue affected rumen fermentation in sika deer, significantly increasing the rumen contents of acetic acid, propionic acid, and total volatile fatty acids, and decreasing rumen fluid pH (*p* < 0.05), but had no significant effect on microbial protein or ammoniacal nitrogen content. American ginseng residue also affected the rumen bacterial composition, with significant up-regulation of Bacteroidota abundance in the HGR group, significant increases in Fibrobacterota and *Fibrobacter* abundance in the LGR group, and a significant decrease in *Oscillospiraceae_UCG-005*. Supplementation with ginseng residue had no significant effect on volatile fatty acids in the feces of sika deer, but did affect the composition of fecal bacteria, with significant decreases in Desulfobacterota and *Rikenellaceae_RC9_gut_group* in the HGR group, and a significant increase in *Ruminococcus* in the LGR group (*p* < 0.05). In addition, the abundance of *Paeniclostridium* in the feces decreased linearly with increasing concentration of ginseng residue, with a significant difference among the groups (*p* < 0.05). This study comprehensively evaluated the effects of American ginseng residue as a potential feed additive on the production performance and gastrointestinal bacterial community in antler-bearing sika deer. The results indicated that ginseng residue was a suitable feed additive for improving production performance and health in sika deer.

## 1 Introduction

Ginseng residue is an industrial by-product resulting from the extraction of ginseng plants, such as American ginseng (*Panax quinquefolius* L.) and ginseng (*Panax ginseng* C. A. Mey.). These residues were previously disposed of as landfill, with adverse effects on the ecological environment. Given the efficacy and importance of ginsenosides in human health care products, many residues after ginsenoside extraction still contain active ingredients, such as polysaccharides and amino acids (Sun et al., 2022, 2023); however, extracting these would entail additional costs. Ginseng residue has previously been reported as a novel source of cellulose to provide energy to piglets, after microbial fermentation to break down lignocellulose (Xiao et al., 2022); however, this likely disrupted the functions of the active substances. We therefore considered that it would be possible to avoid wasting the functional ingredients of ginseng residue, such as active polysaccharides, oligopeptides, and sterols, by direct feeding, while simultaneously reducing environmental pressures.

*Panax quinquefolius*, *P. ginseng*, *P. notoginseng*, and *P. japonicus* are all well-known medicinal plants with excellent antioxidant and immunity-boosting effects (Li et al., 2017; Paik et al., 2023), which have different qualities in Chinese medicine and can be used for different diseases (Liu et al., 2020). Its main medicinal components are saponins and polysaccharides (Xiong et al., 2020; Qi et al., 2021). American ginseng is currently widely planted globally, with huge production, with the United States, Canada, China, and South Korea among the main production areas (Punja, 2011). The most commonly reported biological functions of American ginseng polysaccharides involve modulation of the inflammatory response and improvement of immune function. Previous studies showed that neutral polysaccharides in American ginseng significantly reduced expression levels of the pro-inflammatory cytokines interleukin (IL)-1β, IL-6, and tumor necrosis factor-α, and inhibited the massive recruitment of neutrophils in the neural thalamus of zebrafish, thus alleviating inflammation (Xie et al., 2023). Another oligopolysaccharide isolated from American ginseng, CVT-E002, acted on B-lymphocytes and stimulated the proliferation of splenocytes and the production of immunoglobulin (Ig) G in mice (Wang et al., 2001). Three acidic polysaccharides in American ginseng, PPQA2, PPQA4, and PPQA5, also showed immunostimulatory functions (Wang et al., 2015), while the polysaccharide WQP significantly ameliorated antibiotic-induced diarrhea in rats by inhibiting the mitogen-activated protein kinase inflammatory signaling pathway, decreasing inflammatory cytokine levels and the infiltration of inflammatory cells in the ileum and colon, and increasing short-chain fatty acids by increasing the abundance of beneficial bacteria, such as *Lactobacillus* and *Bacteroides intestinalis*, which promote restoration of the intestinal barrier (Ren et al., 2022). In addition to saponins and polysaccharides, protopanaxadiol in American ginseng significantly inhibited the growth of cancer cells by acting on signaling pathways such as TRAIL (Zhang et al., 2015). Some small molecule oligopeptides in American ginseng were shown to increase the activity of oxide-producing dismutase and glutathione peroxidase, reduce malondialdehyde content, and increase expression levels of nuclear respiratory factor 1 and mitochondrial transcription factor A in mice, thus enhancing the body’s scavenging function of reactive oxygen species and relieving fatigue (Li D. et al., 2018). These results indicate that saponin-extracted American ginseng residue retains medicinal value.

Sika deer are an important component of China’s livestock industry, providing humans with high-quality medicinal and edible products such as antlers, blood, and meat; notably however, nutritional research in sika deer is lacking (Bao et al., 2021; Zhou Z. et al., 2022; Takeda et al., 2023). The antler provide a traditional, valuable, and expensive medicinal product with a variety of effects, such as improving sexual performance in mice and boosting immunity (Zang et al., 2016; Xia et al., 2022). Antler production is thus one of the most important stages in the breeding of sika deer, with distinct economic impacts. Sika deer are sensitive and timid by nature and are thus susceptible to stress caused by frequent human interventions in captivity and the inability to change their living environment in response to changes in the natural environment (Zidon et al., 2009). During the antler-growth period, deer also need to cope with high energy and protein requirements, as well as adapting to rapid changes in hormone levels (Price and Allen, 2004; Bartos et al., 2009; Bao et al., 2021).

The effect of American ginseng residue in sika deer farming is unknown. We therefore comprehensively assessed the effect of American ginseng residue on the production performance, digestibility of nutrients, serum biochemical indexes, and gastrointestinal bacteria and bacterial fermentation in antler-bearing sika deer, with the aim of providing a reference for the reuse of waste and the development of novel feed additives.

## 2 Materials and methods

### 2.1 Animal ethics statement

All animal procedures were approved and authorized by the Animal Ethics Committee of the Institute of Special Animal and Plant Sciences, Chinese Academy of Agricultural Sciences (NO. ISAPSAEC-2021-59D).

### 2.2 Experimental material

American ginseng residue is a by-product of ethanol extraction of saponins. The ginseng residue used in these experiments was purchased from Jilin Hongjiu Bio-technology Co. (Tongliao, Jilin, China). The average polysaccharide content of this batch of residue was 31.61%, and no total ginsenosides were detected in the laboratory.

### 2.3 Experimental design and animal management

Twelve healthy 4-year-old male sika deer with similar body weights and antler-regeneration times were selected for this experiment. Each deer was housed in a separate enclosure and assigned randomly to one of three groups fed basal diets supplemented with 0, 1, and 3% American ginseng residue, respectively. The entire experiment lasted for 5 weeks, including a 1-week pre-experimental period to determine the maximum feed intake of the animals and acclimatize them to the diet, followed by a 4-week experimental period. Throughout the 5-week period, the animals were fed at 4 am and 5 pm each day with a total of 2.8 kg of diet (air-dried basis), during which time the animals had free access to water and the pens were cleaned regularly to ensure a clean environment. All the feed provided was consumed by the animals. The composition and nutrient content of the basal diet are shown in Table 1. The experiment was carried out in Shuangyang District, Changchun City, Jilin Province, China (longitude: 125.724, dimension: 43.539, temperature: 19–28°C, wind scale: <3).

**Table 1:** Basic dietary composition and nutrient levels (dry-matter basis).

Ingredients	(g/100 g)	Nutrient levels	(%)
Corn	15.8	GE (MJ/kg)	16.94
Soybean meal	28	DM%	89.62
Wheat bran	6.5	CP%	22.42
Corn gluten feed	4.5	EE%	3.07
DDGS	2.3	NDF%	52.42
Sunflower seed meal	4.6	ADF%	16.71
Expanded urea	0.5		
Soybean oil	0.5		
Bone meal	0.6		
NaCl	0.7		
Premix^1^	1		
Corn yellow silage	35		
Total	100		

DM, dry matter; CP, crude protein; ME, metabolic energy; EE, ether extract; NDF, neutral detergent fiber; ADF, acid detergent fiber. ME was calculated and other values were measured. Premix1: 1 kg of premix contained the following: MgO, 0.076 g; ZnSO_4_.H_2_O, 0.036 g; MnSO_4_.H_2_O, 0.043 g; FeSO_4_.H_2_O, 0.053 g; NaSeO_3_, 0.031 g; vitamin A, 2484 IU; vitamin D, 3496.8 IU; vitamin E, 0.828 IU; vitamin K, 0.23 mg; vitamin B1, 0.092 mg; vitamin B2, 0.69 mg; vitamin B12, 0.00138 mg; folic acid, 0.023 mg; nicotinic acid, 1.62 mg; calcium pantothenate, 1.15 mg; CaHPO_4_, 5.17 g; CaCO_3_, 4.57 g.

### 2.4 Sample collection and measurement

Approximately 200 g of fresh feces were collected from the deer each day before morning feeding for 4 days prior to the end of the experiment. Hair and grit were removed from the collected feces and the feces were sprayed with 10% dilute hydrochloric acid for nitrogen fixation. The samples were then divided into two parts and processed immediately. One part was dried at 65°C to constant weight, pulverized, and passed through a 40-mesh sieve to produce air-dried samples to detect crude protein and so on (Yang L. et al., 2022), and the other was sterilized at 85°C for 2 h, dried at 65°C to constant weight, and sieved through an 18-mesh sieve for fiber determination.

On the last day of the experiment, the deer were anesthetized using an anesthesia gun (xylazine hydrochloride injection, 2 mL/100 kg) prior to the morning feeding, the antlers were cut off completely using a sterilized saw and weighed, and approximately 30 mL of blood was obtained. The blood was centrifuged (1,200 × *g*) and the serum was aspirated and stored at −80°C for later use. A special hose is inserted through the mouth to reach the rumen, and then the rumen fluid is extracted by means of negative pressure. Approximately 200 mL of rumen fluid was obtained from each deer via the rumen, of which the first 100 mL was discarded to avoid salivary contamination (Chang et al., 2022). Approximately 20 g of fresh feces were obtained from the rectum of the deer using sterile disposable gloves. The rumen fluid and feces were transferred quickly to liquid nitrogen and stored at −80°C for further analysis.

#### 2.4.1 Digestibility of nutrients

Crude protein, ether extract, and dry matter were determined as described previously [Association of Official Analytical Chemists (AOAC), 2006] using a fully automated Kjeldahl nitrogen analyzer KDN-520 (Hangzhou Lvbo Instrument Co., Hangzhou, Zhejiang, China) and Soxhlet fat extraction B-811 (Büchi Labortechnik AG., Flawil, Switzerland). Neutral detergent fiber and acid detergent fiber (ADF) were determined using a fiber analyzer ANKOM A2000i (Ankom Technology Co., Macedon, NY, USA) according to the methods (Raffrenato and Van Amburgh, 2011; Barbosa et al., 2015). Acid-insoluble ash was used as an indicator and determined as described by the reference (Prawirodigdo et al., 2021). Apparent digestibility of a nutrient = 100 − (100 × A/A_1_ × B_1_/B), where A is the percentage of 2 mol/L hydrochloric acid-insoluble ash in the sample, A_1_ is the percentage of 2 mol/L hydrochloric acid-insoluble ash in the feces, B_1_ is the percentage of that nutrient in the feces, and B is the percentage of that nutrient in the sample.

#### 2.4.2 Serum biochemical, antioxidant, and immunological indicators

The concentrations of serum triglycerides, total cholesterol, high-density lipoprotein cholesterol, low-density lipoprotein cholesterol, glucose, total protein, albumin, globulin, alkaline phosphatase, and aspartate aminotransferase were analyzed using commercial colorimetric kits (Nanjing Jiancheng Bioengineering Institute, Nanjing, Jiangsu, China) with a Beckman AU480 automatic biochemistry analyzer (Vitalab Selectra E, Spankeren, The Netherlands). Serum concentrations of IgA, IgM, and IgG were quantified using enzyme-linked immunoassay kits (MLBIO, Shanghai, China). Serum antioxidant levels of total superoxide dismutase, glutathione peroxidase, catalase, and total antioxidant capacity were measured using kits (Nanjing Jiancheng Bioengineering Institute, Nanjing, Jiangsu, China), in accordance with the manufacturer’s instructions.

#### 2.4.3 Volatile fatty acids and rumen fermentation

Rumen fluid samples were centrifuged at 5000 × *g* for 10 min at 4°C, and the supernatant was added to 25% metaphosphoric acid solution containing an internal standard at a ratio of 5:1 (supernatant: internal standard), mixed well, frozen at −20°C overnight, centrifuged at 10,000 × *g* for 10 min at 4°C, and the supernatant was prepared. Fecal samples (30 mg) were placed in a glass tube and 900 μL of 0.5% phosphoric acid was added, mixed well, centrifuged at 14,000 × *g* for 10 min at 4°C. An equal amount of ethyl acetate was added to 800 μL of supernatant for extraction, mixed well, and centrifuged at 14,000 × *g* for 10 min at 4°C. The upper layer of the organic phase was mixed with 4-methylglutaric acid and prepared for use. The prepared samples were analyzed by mass spectrometry using an Agilent 7890A/5975C gas-mass spectrometer (Agilent Technologies Inc., Santa Clara, CA, USA). The chromatographic peak areas and retention times were extracted using MSD ChemStation software (Version B.08.00, Agilent Technologies Inc., Santa Clara, CA, USA). Standardized curves were plotted to calculate the contents of volatile fatty acids in the samples (Wang M. et al., 2016; Zhang et al., 2017).

The concentrations of ammoniacal nitrogen and microbial proteins in the rumen fluid were determined using appropriate kits (Nanjing Jiancheng Bioengineering Institute, Nanjing, Jiangsu, China) (Shahinian and Reinhold, 1971; Liu S. et al., 2023) and the pH of the rumen fluid was determined using a PHS-3C pH meter (Shanghai INESA Scientific Institute Co., Shanghai, China) (Kong et al., 2020).

#### 2.4.4 16S rRNA amplicon sequencing

DNA was extracted from the samples using the CTAB method (Minas et al., 2011), tested for purity and concentration, and then diluted to 1 ng/μL using sterile water and used as a template for polymerase chain reaction (PCR) amplification using the following primers: 338 F (5′-ACTCCTACGGGGAGGCAGCA-3′) and 806 R (5′-GGACTACHVGGGTWTCTAAT-3′). PCR products were purified by magnetic beads, and detected by electrophoresis using a 2% agarose gel after mixing in equal amounts according to the PCR product concentration. A TruSeq DNA PCR-Free sample preparation kit (Illumina Inc., San Diego, CA, USA) was used for library construction, and the constructed libraries were quantified by Qubit and Q-PCR, qualified, and analyzed by NovaSeq6000 (Illumina Inc.) for on-line sequencing (Li et al., 2015; Zhang et al., 2023).

### 2.5 Data analysis

The Shannon index, Simpson index, abundance-based coverage estimator index, Chao1 index, and UniFrac distance were calculated using Qiime software (Version 1.9.1). Principal coordinates analysis (PCoA) plots were plotted using R software (Version 2.15.3). PCoA analysis was performed using the WGCNA, stats, and ggplot2 packages in R. Linear discriminant analysis effect size (LEfSe) was performed using the LEfSe package with a default setting of linear discriminant analysis (LDA) score of 3. Adonis analysis was performed using the adonis function in the R vegan package.

Data were analyzed using SPSS software (IBM SPSS Statistics 26; IBM-SPSS Inc., Chicago, IL, USA). The Shapiro–Wilk test was used to determine if the data conformed to a normal distribution, the Kruskal–Wallis test was used to determine non-conformity, and the Bonferroni method was used for multiple comparisons if *p* < 0.05. Normally distributed data were analyzed using parametric tests and the F-test was used to determine variance alignment. One-way ANOVA was used and *post hoc* multiple comparisons were made using Tukey’s test if variance alignment was met, and the *t-*test was used if variance alignment was not met. Values were expressed as mean ± standard error, with a significance value of *p* < 0.05.

## 3 Results

### 3.1 Effect of American ginseng residue on antler production and apparent digestibility of nutrients in sika deer

The addition of American ginseng residue to the diet significantly increased antler production by sika deer during the antler-bearing period (Table 2), with significantly greater production in the HGR group compared with the CON group (*p* < 0.05). Antler production was also increased in the LGR group, but the difference was not significant. The apparent digestibility of the ADF was significantly increased in the LGR and HGR groups compared with the CON group (*p* < 0.05), but there was no significant difference in the apparent digestibility of other nutrients among the groups.

**Table 2:** Antler weight and apparent fecal digestibility of nutrients.

Item (%)	CON	LGR	HGR	*p*-value
Antler weight (g)	931.50^a^ ± 34.77	1115.00^ab^ ± 58.38	1276.30^b^ ± 114.73	0.03
Crude protein	78.64 ± 0.65	80.11 ± 0.88	77.34 ± 0.87	0.13
Ether extract	89.52 ± 3.82	87.66 ± 3.69	86.32 ± 1.20	0.77
Dry matter	73.44 ± 1.12	75.41 ± 2.77	74.81 ± 1.08	0.75
NDF	75.08 ± 0.97	78.70 ± 2.03	76.80 ± 0.74	0.25
ADF	59.54^a^ ± 0.96	67.12^b^ ± 1.33	64.70^b^ ± 1.17	0.01

ADF, acid detergent fiber; NDF, neutral detergent fiber. Means with different lowercase superscript letters significantly different at *p* < 0.05.

### 3.2 Serum biochemical indexes in the three groups of sika deer

The addition of American ginseng residue to the diet had no significant effect on any of the measured serum biochemical indexes (Table 3), and no effect on glycolipid metabolism, protein metabolism, or enzymes related to liver and kidney metabolism (*p* > 0.05). Serum globulin levels tended to increase linearly with increasing ginseng residue concentration, but the difference was not significant (*p* < 0.1).

**Table 3:** Serum biochemical indicators.

Item	CON	LGR	HGR	*P*-value
TG (mmol/L)	0.17 ± 0.01	0.18 ± 0.01	0.19 ± 0.01	0.55
CHO (mmol/L)	1.95 ± 0.03	1.91 ± 0.08	1.93 ± 0.06	0.87
HDL-C (mmol/L)	1.62 ± 0.08	1.55 ± 0.05	1.60 ± 0.06	0.72
LDL-C (mmol/L)	0.27 ± 0.02	0.25 ± 0.02	0.28 ± 0.02	0.66
Glucose (mmol/L)	8.01 ± 0.09	8.38 ± 0.39	8.31 ± 0.44	0.72
TP (g/L)	63.61 ± 0.64	63.55 ± 1.35	63.75 ± 0.87	0.98
ALB (g/L)	35.60 ± 0.54	33.80 ± 1.08	33.75 ± 1.42	0.42
GLB (g/L)	28.01 ± 0.61	29.75 ± 0.45	30.25 ± 0.76	0.07
Urea (mmol/L)	11.65 ± 0.12	10.79 ± 0.70	10.27 ± 0.48	0.20
ALP (U/L)	393.18 ± 4.43	385.35 ± 10.13	388.24 ± 10.44	0.81
AST (U/L)	51.77 ± 2.75	51.17 ± 2.85	52.41 ± 1.69	0.94

TG, triglycerides; CHO, cholesterol; HDL-C, high-density lipoprotein cholesterol; LDL-C, low-density lipoprotein cholesterol; TP, total protein; ALB, albumin; GLB, globulin; AST, aspartate aminotransferase; ALP, alkaline phosphatase.

### 3.3 Effects of American ginseng residue on antioxidants and immune status of sika deer

Supplementation with American ginseng residue improved the immune and antioxidant statuses of sika deer during the antler-bearing period (Table 4). Serum IgA levels increased significantly (*p* < 0.05) and linearly in the LGR and HGR groups compared with the CON group. In addition, catalase and glutathione peroxidase activities increased significantly (*p* < 0.05) in the HGR group and glutathione peroxidase activity increased significantly (*p* < 0.05) in the LGR group compared with the CON group, but there were no significant differences in the other indexes among the three groups.

**Table 4:** Immune and antioxidant indicators.

Item	CON	LGR	HGR	*P*-value
IgA (ug/mL)	490.20^a^ ± 13.74	543.45^b^ ± 18.08	557.70^b^ ± 4.99	0.01
IgG (mg/mL)	3.92 ± 0.06	4.24 ± 0.08	4.12 ± 0.19	0.26
IgM (ug/mL)	103.51 ± 2.43	105.66 ± 3.19	105.59 ± 2.25	0.81
CAT (U/mL)	2.72^a^ ± 0.05	2.93^ab^ ± 0.09	3.18^b^ ± 0.14	0.04
GSH-PX (U/mL)	168.17^a^ ± 3.64	182.26^b^ ± 2.19	182.42^b^ ± 4.47	0.03
T-SOD (U/mL)	101.10 ± 2.46	103.94 ± 4.06	104.18 ± 6.57	0.77
T-AOC (U/mL)	1.13 ± 0.10	1.23 ± 0.12	1.15 ± 0.11	0.82

Ig, immunoglobulin; CAT, catalase; GSH-PX, glutathione peroxidase; T-SOD, total superoxide dismutase; T-AOC, total antioxidant capacity. Means with different lowercase superscript letters were significantly different at *p* < 0.05.

### 3.4 Supplementation of American ginseng residue altered rumen fermentation and bacterial communities in sika deer

We compared the rumen fermentation parameters among the three groups of sika deer (Table 5), and found that the acetate content was significantly higher in the LGR group compared with the CON group, and the propionate content was significantly higher in the LGR and HGR groups (*p* < 0.05). In addition, the total volatile fatty acid content was also significantly higher in the LGR group than in the CON group. The ammonia nitrogen and microbial protein contents of the rumen fluid were similar in all three groups of sika deer (*p* > 0.05), but the rumen fluid pH was significantly lower in the HGR group compared with the CON group (*p* < 0.05).

**Table 5:** Indicators of rumen fermentation and volatile fatty acids in rumen fluid.

Item	CON	LGR	HGR	*P*-value
Acetate (mmol/L)	54.87^a^ ± 1.75	61.62^b^ ± 0.65	57.94^ab^ ± 1.85	0.03
Propionate (mmol/L)	13.93^a^ ± 0.48	17.16^b^ ± 0.45	17.46^b^ ± 1.04	0.01
Isobutyrate (mmol/L)	1.56 ± 0.15	1.32 ± 0.10	1.51 ± 0.17	0.50
Butyrate (mmol/L)	6.18 ± 0.29	6.24 ± 0.51	7.13 ± 0.51	0.30
Isovalerate (mmol/L)	1.71 ± 0.07	1.29 ± 0.20	1.70 ± 0.19	0.19
Valerate (mmol/L)	0.40 ± 0.02	0.48 ± 0.01	0.47 ± 0.05	0.31
TVFAs (mmol/L)	78.65^a^ ± 2.23	88.11^b^ ± 1.16	86.21^ab^ ± 3.09	0.04
Ammonia (mg/dL)	10.99 ± 0.41	10.34 ± 0.30	10.15 ± 0.50	0.37
Microbial proteins (mg/mL)	1.88 ± 0.08	1.96 ± 0.10	1.98 ± 0.08	0.72
Rumen fluid pH	7.03^b^ ± 0.07	6.86^ab^ ± 0.06	6.78^a^ ± 0.01	0.04

TVFAs, total volatile fatty acids. Means with different lowercase superscript letters were significantly different at *p* < 0.05.

**Figure 1:**
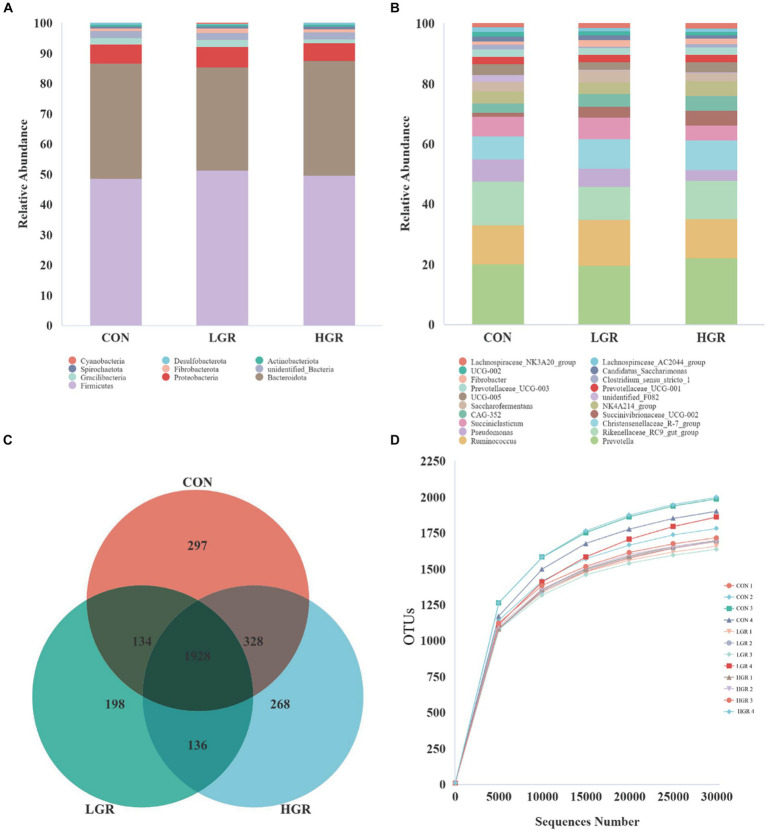
Abundances of top 10 bacteria at phylum level **(A)** and top 20 at genus level **(B)** in the rumen. Venn diagrams **(C)** and dilution curves **(D)** of rumen bacteria.

Illumina Nova sequencing, construction of PCR-free libraries, followed by paired-end sequencing. After splicing the reads, an average of 85,245 tags were measured per sample, and an average of 84,552 valid data were obtained after quality control, with the amount of valid data for quality control amounting to 65,081, and the effective quality control rate of 76.42%. The sequences were clustered into operational taxonomic units (OTUs) with 97% concordance. A total of 4,869 OTUs were obtained, and 3,289 OTUs were filtered after removing data for Archaea, unknown, and no blast hits. Among these, 1,928 OTUs were common to all three groups of Figure 1C. The sequences of the OTUs were then annotated for species using the Silva138 database. At the phylum level, Bacillota and Bacteroidota were the dominant rumen bacteria in sika deer, accounting for 47.5 and 35.5% of the total bacterial abundance, respectively (Figure 1A). At the genus level, *Prevotella*, *Ruminococcus*, *Rikenellaceae_RC9_gut_group*, *Pseudomonas*, *Christensenellaceae_R-7_group*, and *Succiniclasticum* were the dominant genera (Figure 1B), accounting for about half of all genera. We plotted a curve by randomly selecting the amount of sequencing data for the sample and its corresponding number of species to obtain the dilution curve of the sample (Figure 1D); the curve gradually flattened out and the sequencing data were reasonable.

**Figure 2:**
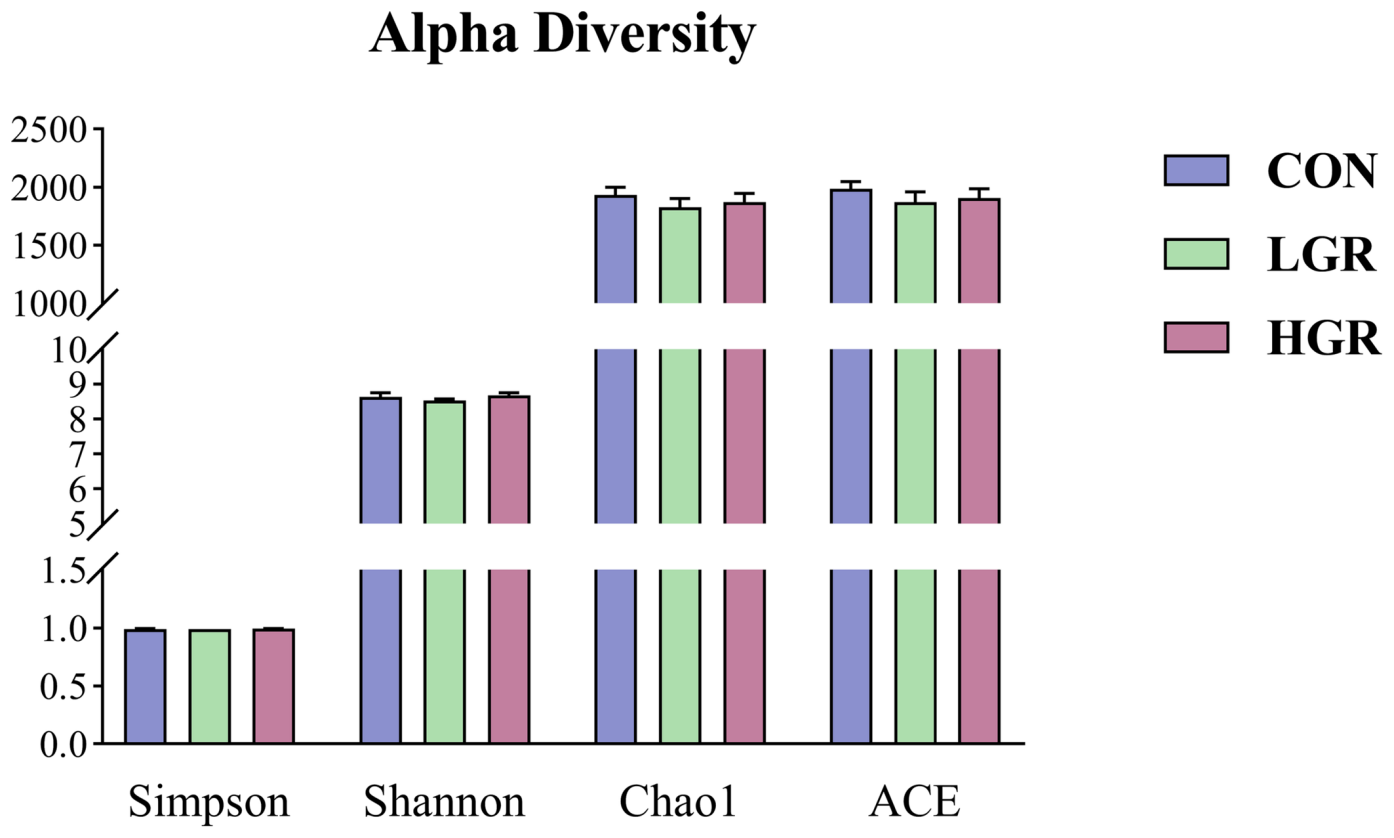
Bacterial alpha diversity in the rumen of sika deer.

**Figure 3:**
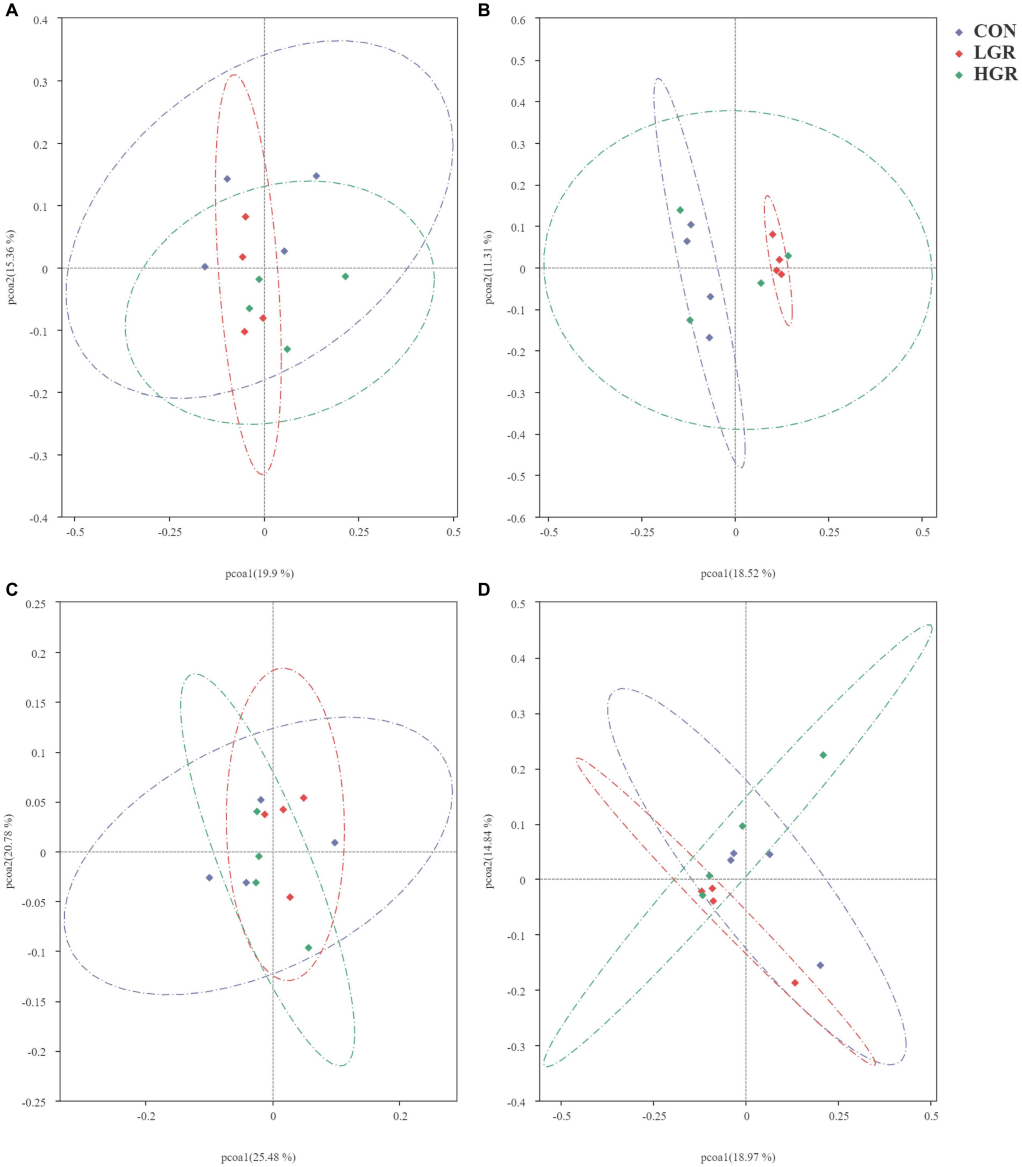
Principal co-ordinates analysis of bacterial communities in the rumen of sika deer based on Bray–Curtis distance **(A)**, binary-Jaccard distances **(B)**, weighted-UniFrac distance **(C)**, and unweighted-UniFrac distance **(D)**.

**Figure 4:**
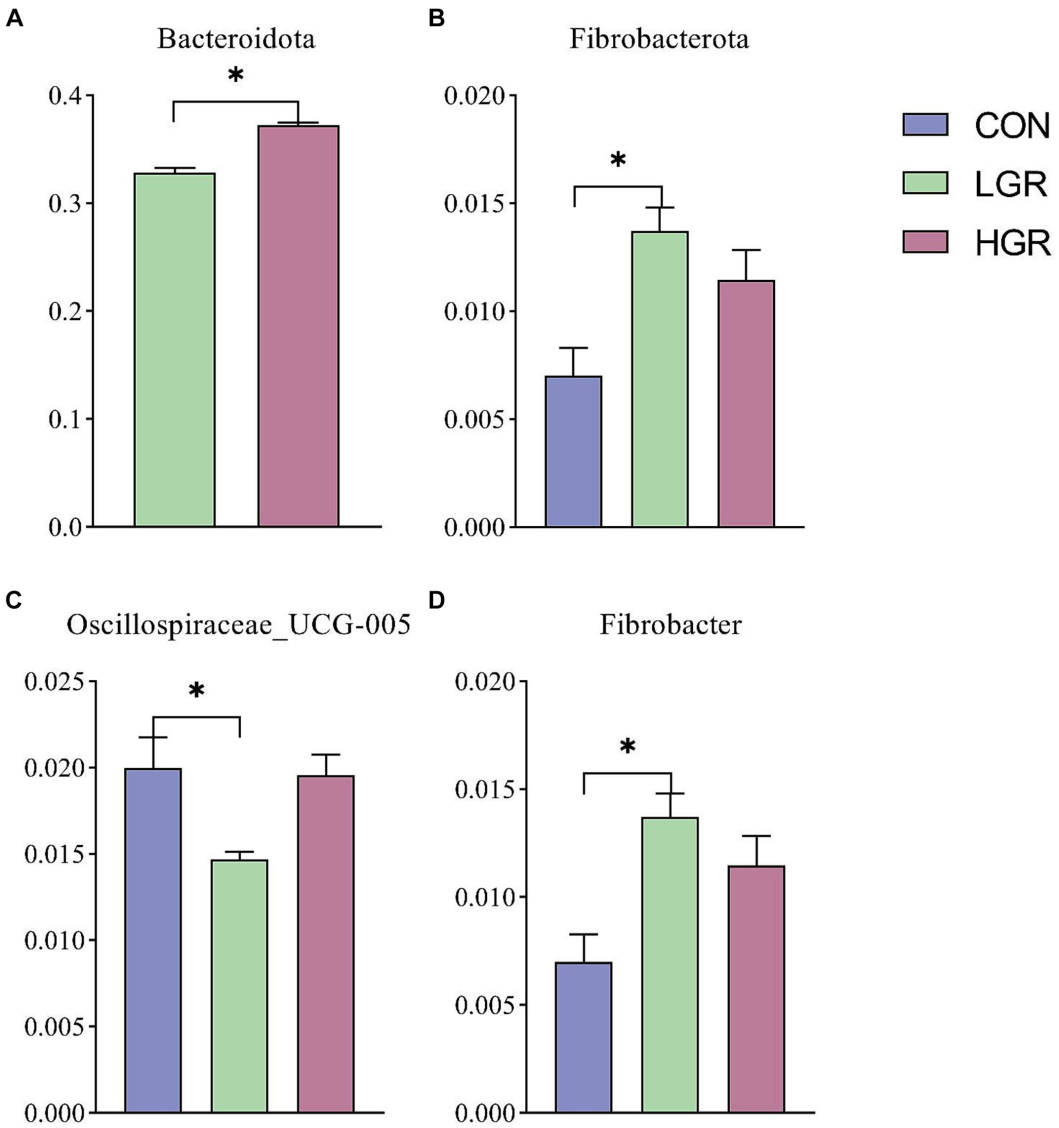
Differences in rumen bacteria at phylum level by *t*-test **(A)** and ANOVA **(B)**. Differences in rumen bacteria at genus level **(C,D)**. *p < 0.05.

**Figure 5:**
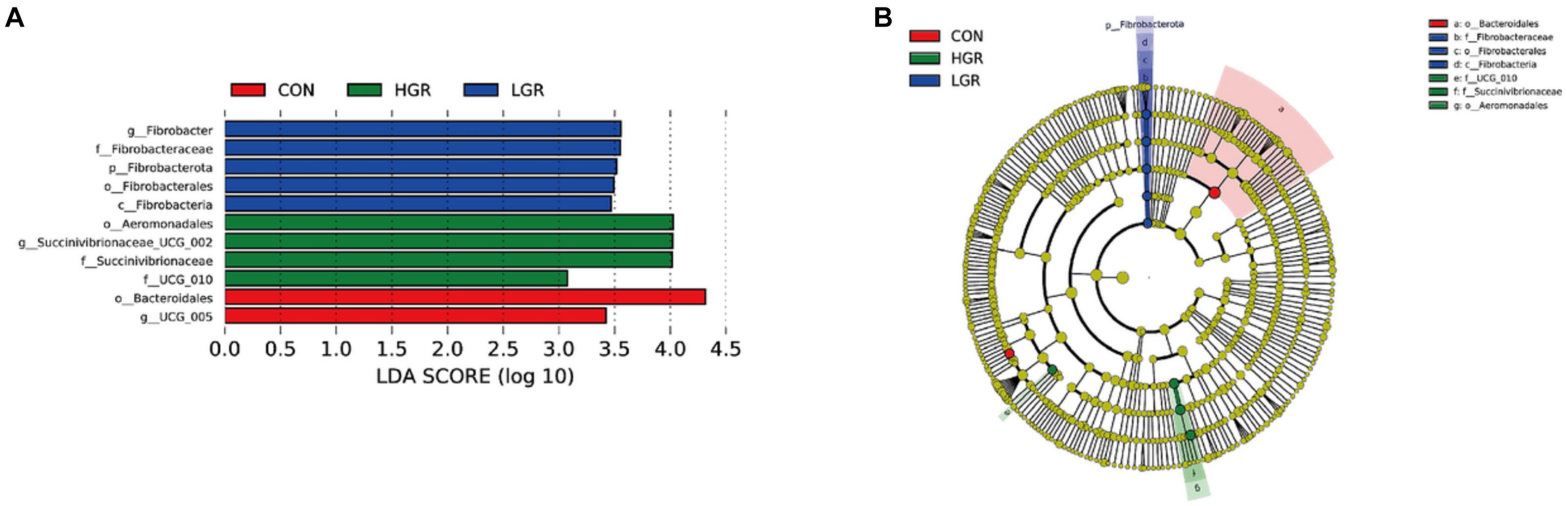
Linear discriminant analysis (LDA) effect size (LEfSe) analysis of rumen bacteria in sika deer. **(A)** LDA score of rumen microbiota (score > 3 significant). **(B)** Cladogram of LEfSe showing nodes of significant bacteria on evolutionary branches. Yellow nodes, no difference; other colored nodes, significant differences.

We investigated the effect of American ginseng residue on the abundance and diversity of rumen bacterial communities by comparing the alpha diversities among the three groups. There were no significant differences (*p* > 0.05) in the Shannon index, Simpson index, Chao1 index, and ACE index of the rumen bacteria (Figure 2). Based on the Bray–Curtis, binary-Jaccard, weighted UniFrac, and unweighted UniFrac distances, PCoA (Figure 3) and adonis analysis (Table 6) showed that the bacterial compositions of the CON and LGR groups were significantly separate (binary-Jaccard distances: *p* < 0.05, [CON vs. LGR]). We further compared bacteria with significantly different abundances at the phylum and genus levels. At the phylum level, Bacteroidota were significantly more abundant in the HGR group than in the LGR group (Figure 4A) and Fibrobacterota were significantly more abundant in the LGR group compared with the CON group (Figure 4B). At the genus level, *Oscillospiraceae_UCG-005* were significantly less abundant in the LGR group (Figure 4C) and *Fibrobacter* were significantly more abundant in the LGR group compared with the CON group (Figure 4D). LDA showed that 11 bacterial species differed significantly from phylum level to genus level in the rumen fluid of the three groups (Figure 5A), Fibrobacteria, Fibrobacterales, and Fibrobacteraceae were significantly enriched under the same branch of the developmental tree in the LGR group, and Aeromonadales and Succinivibrionaceae were enriched in the HGR group under the same branch, while Oscillospirales__UCG-010 and Bacteroidales in the CON group were enriched on different branches (Figure 5B).

**Table 6:** Adonis analysis of bacterial communities in the rumen.

Group	Bray-Curtis		Binary-Jaccard		Weighted-UniFrac		Unweighted-UniFrac	
	*R*^*2*^	*P*	*R*^*2*^	*P*	*R*^*2*^	*P*	*R*^*2*^	*P*
CON VS. LGR	0.180	0.087	0.228	0.029	0.194	0.169	0.166	0.153
CON VS. HGR	0.172	0.193	0.140	0.502	0.130	0.556	0.142	0.443
LGR VS. HGR	0.181	0.053	0.166	0.146	0.209	0.087	0.151	0.410

### 3.5 Effect of American ginseng residue on fecal volatile fatty acids and bacterial communities in sika deer

Fecal volatile fatty acids can reflect bacterial fermentation in the intestine. The feces contents of acetate, propionate, isobutyrate, butyrate, isovalerate, valerate, caproate, and total volatile fatty acids were similar in the three groups of deer (*p* > 0.05) (Table 7).

**Table 7:** Fecal volatile fatty acids.

Item (ug/g)	CON	LGR	HGR	*P*-value
Acetate	1187.36 ± 103.95	1120.14 ± 103.22	1166.37 ± 105.28	0.89
Propionate	404.54 ± 18.81	387.03 ± 15.04	433.68 ± 21.46	0.25
Isobutyrate	29.93 ± 1.29	27.67 ± 2.75	25.85 ± 2.73	0.50
Butyrate	341.07 ± 23.15	327.42 ± 18.43	331.36 ± 18.29	0.88
Isovalerate	17.67 ± 1.48	18.03 ± 3.14	14.68 ± 1.73	0.53
Valerate	56.34 ± 3.96	52.87 ± 3.39	57.69 ± 5.66	0.74
Caproate	3.54 ± 0.16	3.40 ± 0.23	4.06 ± 0.44	0.31
TVFAs	2040.47 ± 137.26	1936.58 ± 105.39	2033.71 ± 137.90	0.81

TVFAs, total volatile fatty acids.

**Figure 6:**
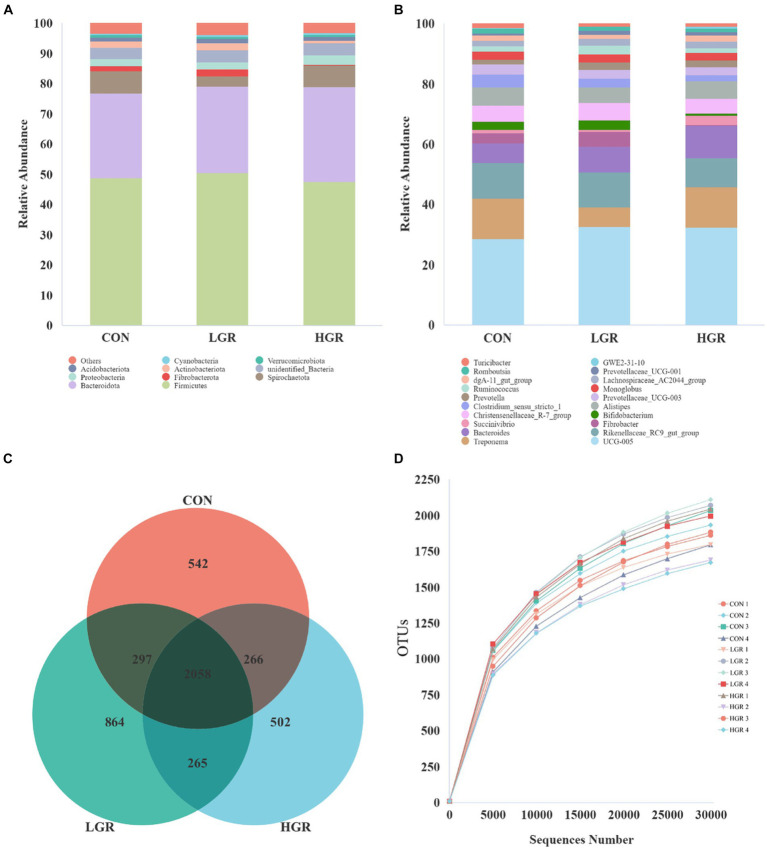
Abundance of top 10 bacteria in feces at phylum level **(A)** and top 20 at genus level **(B)**. Venn diagrams **(C)** and dilution curves **(D)** of fecal bacteria.

The fecal samples were sequenced and an average of 73,449 tags were measured for each sample. 73,020 data were obtained on average after quality control, and the amount of valid data for quality control amounted to 55,270, and the effective rate of quality control was 75.25%. A total of 5,981 OTUs were obtained after clustering with 97% concordance, and 4,794 OTUs were obtained after screening, of which 2,058 OTUs were common to all three groups (Figure 6C). The dominant bacteria in feces at the phylum level were Bacillota and Bacteroidota, which accounted for 48.6 and 29.2% of the total abundance, respectively (Figure 6A). The dominant bacteria at the genus level were *Oscillospiraceae_UCG-005* (16.1%), *Treponema* (5.7%), *Rikenellaceae_RC9_gut_group* (5.7%), and *Bacteroides* (4.3%) (Figure 6B). The dilution curve gradually flattened out (Figure 6D) and the data were reasonable.

**Figure 7:**
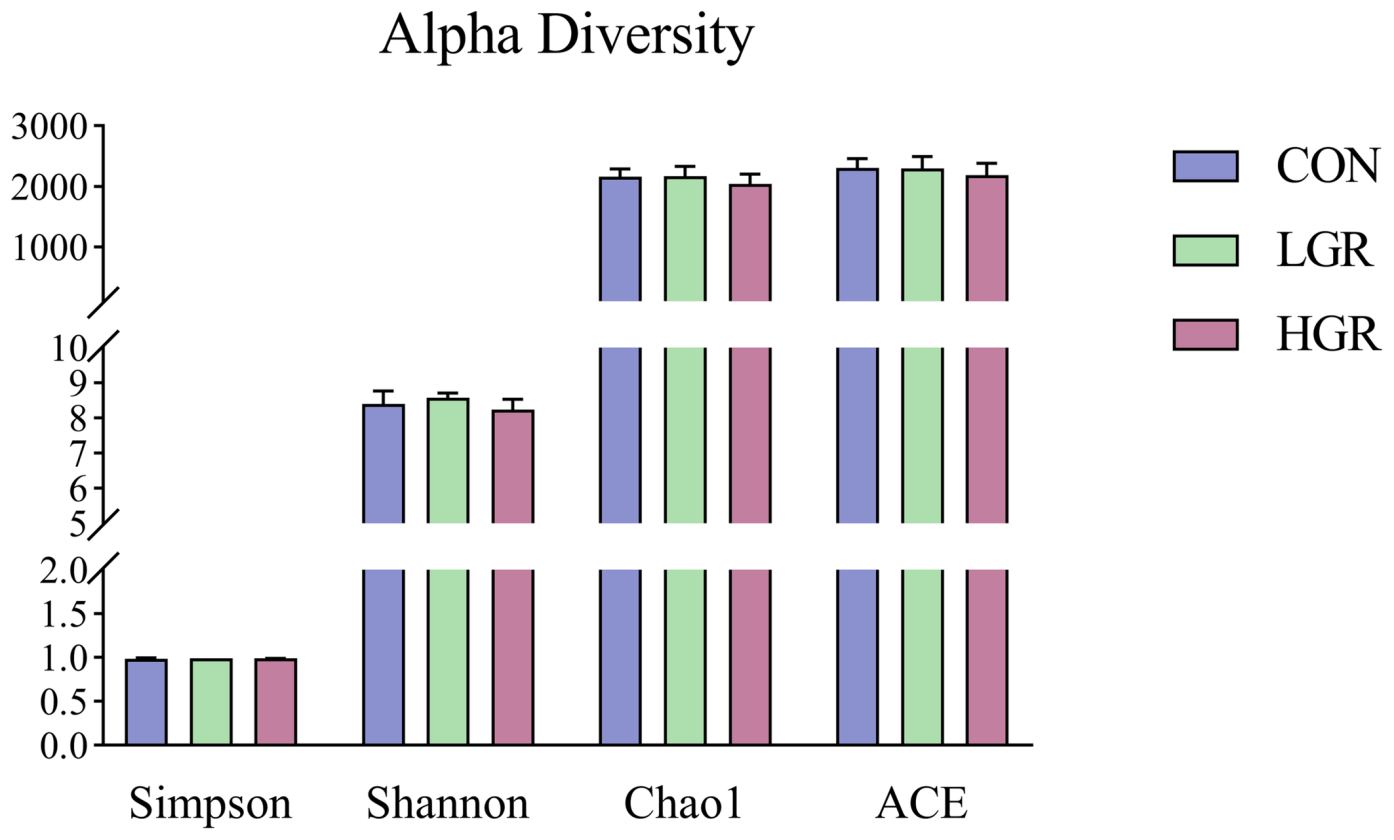
Bacterial alpha diversity in the feces of sika deer.

The fecal alpha diversity results were similar for all three groups (*p* > 0.05) (Figure 7).

**Figure 8:**
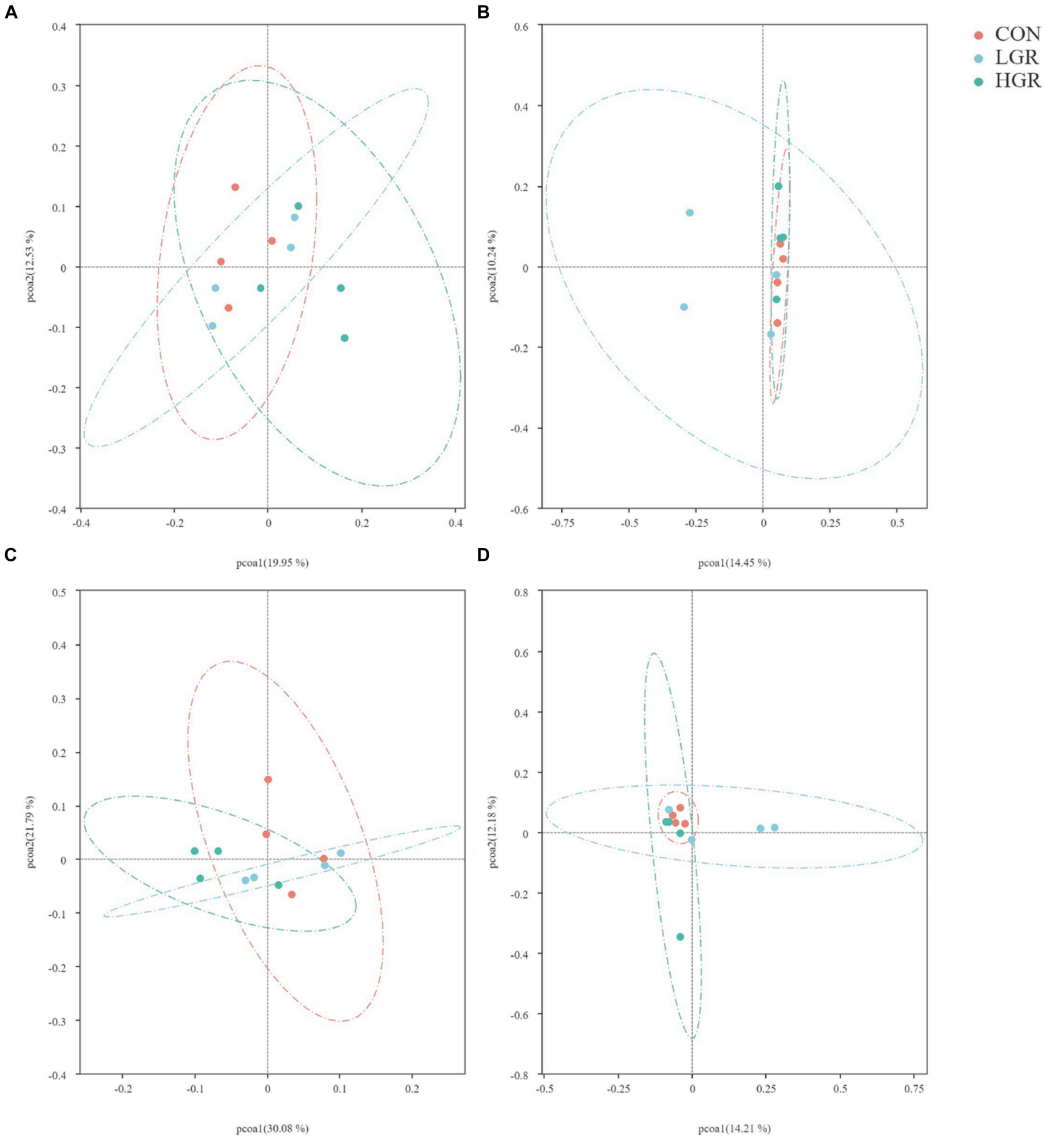
Principal co-ordinates analysis of bacterial communities in the feces of sika deer based on Bray–Curtis distance **(A)**, binary-Jaccard distances **(B)**, weighted-UniFrac distance **(C)**, and unweighted-UniFrac distance **(D)**.

**Figure 9:**
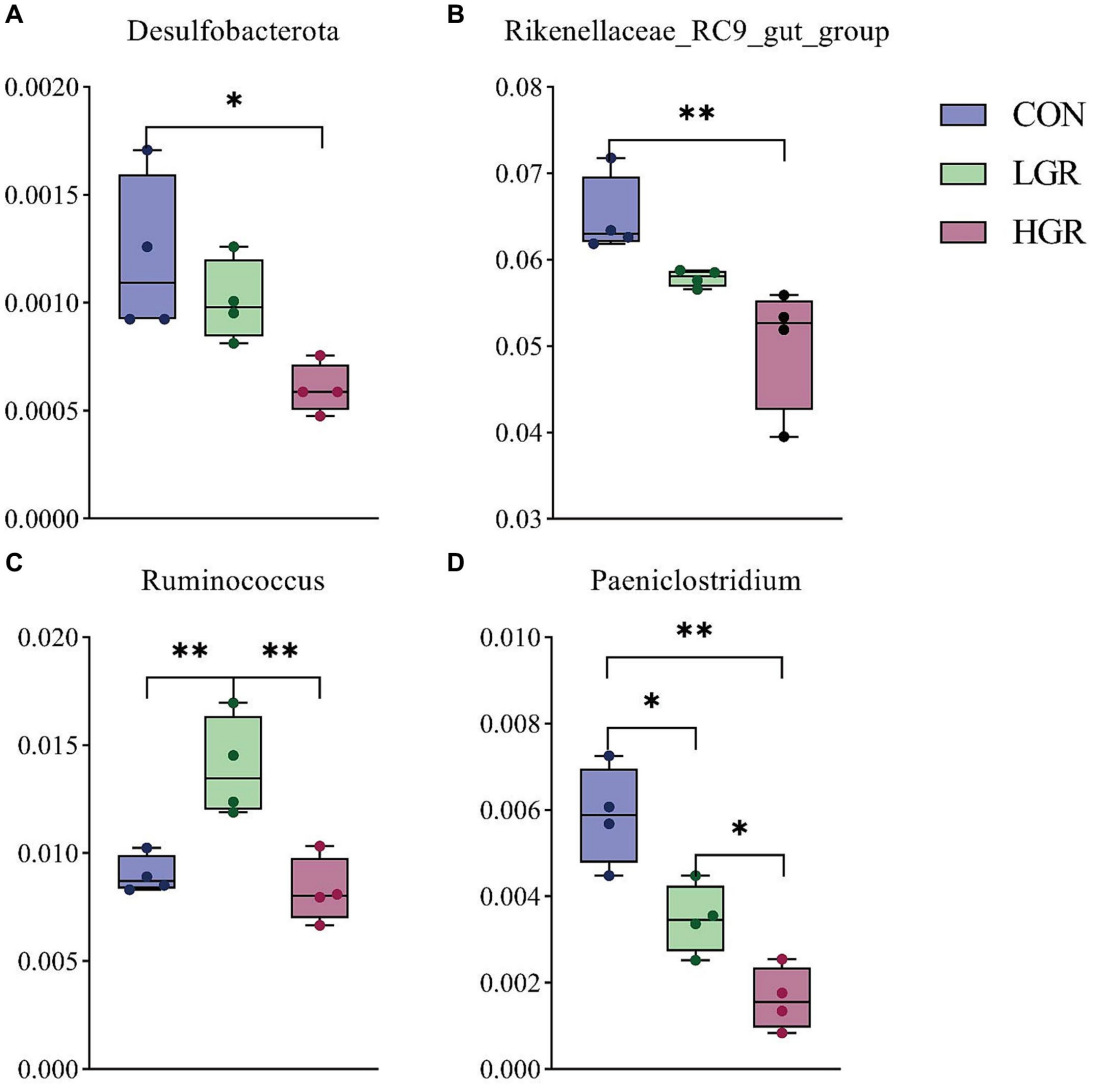
Differences in feces bacteria at phylum level **(A)** and genus level **(B–D)**. *p < 0.05; **p < 0.01.

**Figure 10:**
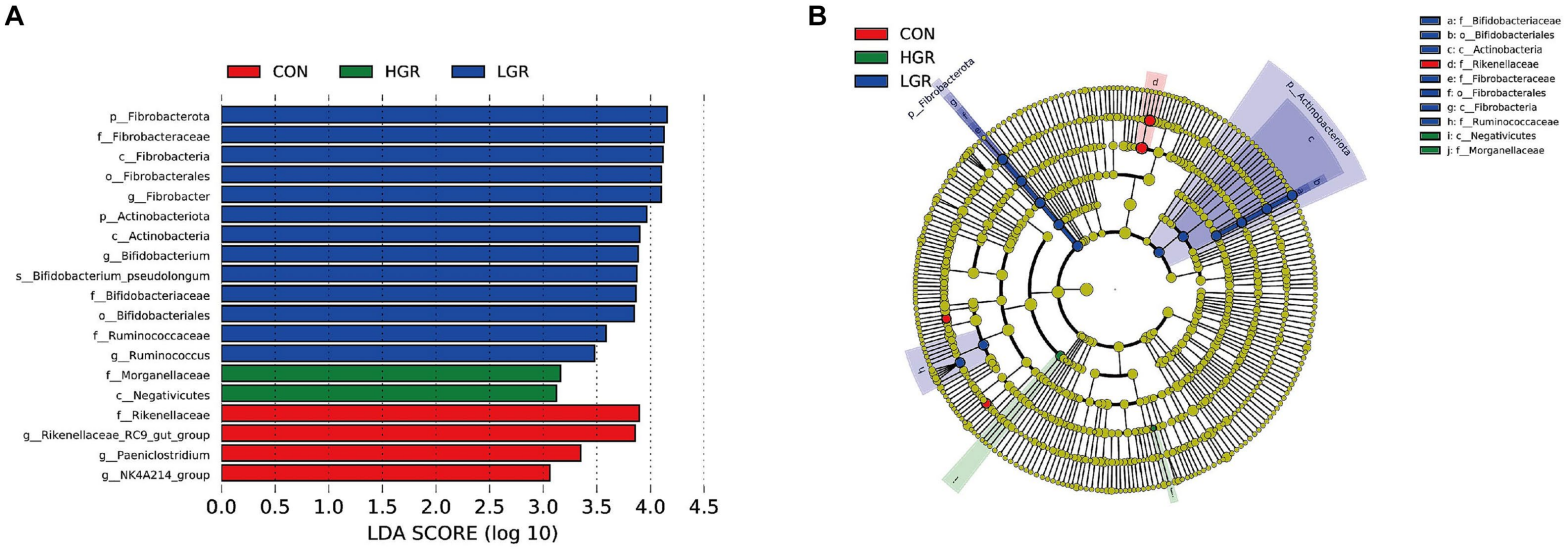
Linear discriminant analysis (LDA) effect size (LEfSe) analysis of fecal bacteria in three groups of sika deer. **(A)** LDA score of fecal microbiota (score > 3 significant). **(B)** Cladogram of LEfSe showing nodes of significant bacteria on evolutionary branches. Yellow nodes, no significant difference; other colored nodes, significant differences.

The results of PCoA (Figure 8) and adonis analysis (Table 8) showed that the fecal bacterial composition differed significantly between the CON and HGR groups, especially in terms of binary-Jaccard and unweighted-UniFrac distances (*p* < 0.05). Further analysis of the differential bacteria in the feces of the three groups (Figure 9) showed a significant decrease in Desulfobacterota abundance at the phylum level in the HGR group compared with the CON group (*p* < 0.05). At the genus level, the abundance of *Rikenellaceae_RC9_gut_group* was highly significantly decreased in the HGR group compared with the CON group (*p* < 0.01), and the abundance of *Ruminococcus* was highly significantly increased in the HGR group compared with the CON and HGR groups (*p* < 0.01), while the abundance of *Paeniclostridium* declined linearly in line with increasing addition of ginseng residue, with significant differences among the groups (*p* < 0.05). LDA (Figure 10A) showed significant enrichment from the phylum to the genus level for 19 bacterial species in the three groups, with Fibrobacterota providing the greatest contribution to the evolutionary tree (Figure 10B) in the LGR group. Fibrobacterota, Fibrobacteria, Fibrobacterales, Fibrobacteraceae, and *Fibrobacter* were enriched in the same branch, and Actinobacteriota, Actinobacteria, Bifidobacteriales, Bifidobacteriaceae, and *Bifidobacterium* were also equally enriched in the same branch, in addition to homology between Rikenellaceae and *Rikenellaceae_RC9_gut_group* in the CON group.

**Table 8:** Adonis analysis of bacterial communities in feces.

Group	Bray-Curtis		Binary-Jaccard		Weighted-UniFrac		Unweighted-UniFrac	
	*R*^*2*^	*P*	*R*^*2*^	*P*	*R*^*2*^	*P*	*R*^*2*^	*P*
CON VS. LGR	0.132	0.738	0.167	0.062	0.131	0.558	0.166	0.056
CON VS. HGR	0.199	0.07	0.157	0.024	0.237	0.093	0.154	0.029
LGR VS. HGR	0.163	0.208	0.165	0.066	0.222	0.107	0.162	0.095

## 4 Discussion

American ginseng and sika deer are specific agricultural and animal husbandry industries, respectively, in Northeast China (Li et al., 2014), and the application of American ginseng by-products in sika deer breeding can thus maximize local advantages with mutual benefits. However, evidence for the role of American ginseng residue in the production of antler-bearing sika deer is currently lacking. Previous studies showed that animals experienced stress during specific physiological phases due to environmental changes or rapid changes in their own hormone levels, potentially leading to gastrointestinal tract damage, redox imbalance, and microflora dysbiosis, and ultimately affecting the performance and health of the animal (Campbell et al., 2013; Gresse et al., 2017; Deng et al., 2020). The current results showed that dietary supplementation with American ginseng residue significantly increased antler production as a direct result of improved utilization of cellulose in the feed, possibly related to the amelioration of gut damage and restoration of function. Previous studies found that American ginseng polysaccharides effectively treated intestinal mucosal injuries by up-regulating the ratio of villus height to crypt depth, increasing the number of cuprocytes and the expression of tight junction proteins (Zhou et al., 2021; Ren et al., 2022). In a mouse model of dextran sodium sulfate-induced enteritis, American ginseng extract reduced ileal inflammation and edema, increased villus length to alleviate diarrhea symptoms, and was effective in both prophylactic and therapeutic treatments (Jin et al., 2008; Wang C. Z. et al., 2016). Differences in serum activities and metabolite contents of various enzymes among the three subgroups in the present study demonstrated further the effects of ginseng residue in sika deer. Serum levels of various enzymes and transport products related to hepatic and renal metabolism were unaffected by the residue, indicating that the drug components in the residue did not affect hepatic and renal functions. Notably however, diet supplementation with American ginseng residue significantly enhanced the activities of catalase and glutathione peroxidase, which play key antioxidant roles in animals. Catalase is found widely in living organisms, and its main role is to scavenge the strongly cytotoxic hydrogen peroxide produced by the body’s metabolism (Alfonso-Prieto et al., 2009). Glutathione peroxidase exerts its antioxidant function by catalyzing the reduction of hydrogen peroxide by glutathione to achieve the scavenging of oxygen free radicals, and the oxidized glutathione generated can be reduced by other enzymes to play a role in the glutathione cycle (Arthur, 2000). In addition, serum IgA levels were significantly increased in the LGR and HGR groups, consistent with previous reports that American ginseng polysaccharides and other components exerted various effects to achieve immune-enhancing effects, such as activation of macrophages and enhanced phagocytosis (Yu et al., 2014), promotion of lymphocyte proliferation to increase Ig expression (Yu et al., 2017), and activation and inhibition of inflammatory responses (Jin et al., 2008; Yang S. et al., 2022). It mainly acts through binding to Toll-like receptors 2 ad 4 (Yin et al., 2019), to achieve the activation and recruitment of IL receptor-related kinases and phosphorylation of various immune-response-related protein kinases, ultimately resulting in immunomodulatory effects (Hoshino et al., 1999; Liu et al., 2017). In addition, American ginseng polysaccharides bind to complement receptor CR3, C-type lectin receptor, and scavenger receptor to regulate the immune response (Loh et al., 2017; Prado et al., 2020; Su et al., 2020). Polysaccharides and other active ingredients in ginseng dregs thus have good antioxidant and immunomodulatory effects that may improve the utilization of nutrients and the overall health of sika deer.

Ruminant diets contain a large proportion of crude feed components that are difficult to digest, and they thus rely on microorganisms in the rumen to ferment and degrade the cellulose and hemicellulose, which can produce large amounts of short-chain fatty acids (Wang et al., 2018; Liang et al., 2022), which then enter the animal’s body via the gastrointestinal tract epithelial cells and serve as an important source of energy. The composition of rumen microorganisms thus has an important impact on the animal (Baldwin and Connor, 2017). The current results showed that Bacillota and Bacteroidota dominated the rumen in sika deer at the phylum level, while *Prevotella*, *Ruminococcus*, and *Rikenellaceae_RC9_gut_group* were the dominant bacteria at the genus level, in agreement with previous studies of rumen bacteria in sika deer (Li et al., 2013, 2015; Si et al., 2021). The results of β-diversity analysis indicated that dietary supplementation with American ginseng residue resulted in significant differences in rumen bacterial composition between the LGR and CON groups, with significant differences at the phylum and genus levels for Fibrobacterota, *Oscillospiraceae_UCG-005*, and *Fibrobacter*. Fibrobacterota is an important bacterial phylum that degrades cellulose in the gastrointestinal tract of herbivores and provides an important physiological basis for the adaptation of animals to harsh dietary conditions (Ransom-Jones et al., 2012; Jewell et al., 2013). In addition to being present in ruminants, this group of bacteria is also widely present in the intestinal tracts of termites, camels, and other animals that rely heavily on cellulose-based diets (König et al., 2013; Gharechahi et al., 2022; Zhao et al., 2023). *Fibrobacter*, a genus of Fibrobacterota, has likewise been reported as an important cellulose-degrading bacterium (Béra-Maillet et al., 2004). The current results thus explain the significantly higher apparent digestibility of ADF in the LGR group compared with the CON group, as well as the differentially increased levels of acetic acid and propionic acid in the rumen fluid in the LGR and HGR groups. In the same way, red ginseng polysaccharides have a positive effect on the production of short-chain fatty acids in the cattle rumen (Ju et al., 2024). *Oscillospiraceae_UCG-005* is also a class of cellulose-degrading bacteria in the rumen (Li et al., 2021), but is also thought to play a role in the catabolism and utilization of dietary lipids, with significant increases in abundance, especially in animals exposed to high-fat diets (Liu Y. et al., 2023; Wu Z. L. et al., 2023). This is consistent with the results of a decrease in apparent digestibility of ether extracts in the three groups of deer, although the difference was not significant. In addition, deer in the HGR group showed a significant decrease in rumen fluid pH, which may imply an effect on the homeostasis of the rumen internal environment. However, it remained within the healthy range according to previous reports (Zhou X. et al., 2022), and the decrease in rumen fluid pH within the appropriate range was more favorable for the transport of short-chain fatty acids (Yan et al., 2014). Although American ginseng residue reduced the abundance of some beneficial bacteria in the rumen of sika deer, the overall effect on rumen function was positive.

Unlike the rumen microbiome, gut microbes are essential for the survival of all animals. Gut microbes are involved in the digestion and absorption of nutrients in the host (Zhao et al., 2019), influence host growth and development (Sommer and Bäckhed, 2013), participate in the onset and progression of disease (Hsu and Schnabl, 2023), can even affect the host’s mood and sleep (Li Y. et al., 2018). Microorganisms interact with the host via mediators, including metabolites such as short-chain fatty acids, bile acids, and endogenous cannabinoids (de Vos et al., 2022). The dominant bacteria at the phylum level in the intestine in sika deer were Bacillota and Bacteroidota, and the most abundant genera were *Oscillospiraceae_UCG-005*, followed by *Treponema* and *Rikenellaceae_RC9_gut_group*, with clear inconsistency between the dominant bacteria in the intestines and rumen, in agreement with previous reports (Wu Y. et al., 2023). Diversity analysis and adonis analysis showed that the composition of gut bacteria in the HGR group was significantly different from the other two groups, specifically in terms of Desulfobacterota at the phylum level, and *Rikenellaceae_RC9_gut_group*, *Ruminococcus*, and *Paeniclostridium* at the genus level. Previous studies demonstrated that Desulfobacterota were mainly concentrated in the hindgut of animals and were significantly enriched in a wide range of diet-induced intestinal inflammation conditions in mice (Panah et al., 2023; Ruan et al., 2023). They have also been suggested to be a marker of intestinal barrier damage (Rao et al., 2021), largely due to the fact that Desulfobacterota lipopolysaccharides are severe inflammatory stimulants for the host (Huang et al., 2021). *Rikenellaceae_RC9_gut_group* is a beneficial bacterium in the gastrointestinal tract of animals, involved in the fermentation of cellulose and capable of producing short-chain fatty acids (Xi et al., 2023). *Ruminococcus* is a common bacterium in the feces of ruminants, mainly colonizing the jejunum and ileum of the intestinal lumen (Guerra et al., 2022; Zhang et al., 2022), and is involved in the degradation of starch and other complex polysaccharides (Rangarajan et al., 2022). *Ruminococcus* has also been well-documented as an inducer of active inflammation in animals (Hall et al., 2017; Henke et al., 2021), and has been shown to play a role in the maintenance of homeostasis by acting as a key symbiotic component of the intestinal ecosystem (La Reau and Suen, 2018; Crost et al., 2023; Juge, 2023). *Paeniclostridium* is a pathogenic bacterium (DeCandia et al., 2023) that induces inflammation in the intestinal tract and in various tissues in animals (Nyaoke et al., 2020; Gryaznova et al., 2021), via its ability to produce cytotoxic endotoxins (Watts et al., 2019; Li et al., 2022). Consistent with our results, these studies demonstrated that polysaccharide components contained in American ginseng and red ginseng can effectively regulate the bacterial composition of the intestinal tract of animal animals, significantly increase the abundance of beneficial bacteria and reduce the level of pathogenic bacteria, which is beneficial to the improvement of diarrhea in animals (Sha et al., 2023; Song et al., 2023; Min et al., 2024). We also detected Fibrobacterota in the intestine, and although its abundance did not differ not significantly among the groups, LEfSe analysis revealed that Fibrobacterota and its subordinate multiple bacteria, from the class to the genus level, were significantly enriched, with the highest contribution in the LGR group. This may facilitate the digestion of cellulose in the hindgut, and also suggests that, despite differences in microbial composition between the rumen and intestine (Zou et al., 2020), like communication between the upper and lower intestinal tracts, the effects of changes in the dominant bacteria in the rumen are transmitted to the intestinal tract (Steele et al., 2016); however, it is not known whether this microbial migration and crosstalk can occur in the reverse direction.

## 5 Conclusion

Overall, dietary supplementation with American ginseng residue increased the apparent digestibility of nutrients, improved the immune and antioxidant statuses, and promoted antler production in sika deer during the antler-bearing period, as well as positively regulating the gastrointestinal flora and bacterial fermentation. These results suggest that American ginseng residue may be a suitable feed additive for the production of sika deer.

## Data availability statement

The datasets presented in this study can be found in online repositories. The names of the repository/repositories and accession number(s) can be found at: NCBI – https://www.ncbi.nlm.nih.gov/bioproject/?term=PRJNA1078378.

## Author contributions

YW: Data curation, Writing – original draft, Writing – review & editing. SZ: Data curation, Formal analysis, Writing – review & editing. PZ: Conceptualization, Funding acquisition, Writing – review & editing. HL: Supervision, Writing – review & editing. ZQ: Investigation, Writing – review & editing. WH: Supervision, Writing – review & editing. WY: Visualization, Writing – review & editing. TF: Resources, Writing – review & editing. XZ: Resources, Writing – review & editing. JS: Resources, Writing – review & editing. KW: Conceptualization, Funding acquisition, Resources, Supervision, Writing – review & editing.
